# Gut Microbiota Reshapes the Tumor Microenvironment and Affects the Efficacy of Colorectal Cancer Immunotherapy

**DOI:** 10.1002/cam4.70994

**Published:** 2025-06-18

**Authors:** Fayuan Wang, Weidong Chen, Yingtian Jia, Tao He, Siyi Wu, Jiahui Xiang, Rui Chen, Qingfeng Jiang, Tengjiang Yu, Yong Lan, Wusheng Li, Liang Ma, Ping He, Shichao Li

**Affiliations:** ^1^ The Anorectal Department The Affiliated Traditional Chinese Medicine Hospital, Southwest Medical University Luzhou City Sichuan Province China; ^2^ College of Integrated Traditional Chinese and Western Medicine Southwest Medical University Luzhou City Sichuan Province China; ^3^ The Key Laboratory of Integrated Traditional Chinese and Western Medicine for Prevention and Treatment of Digestive System Diseases of Luzhou City The Affiliated Traditional Chinese Medicine Hospital, Southwest Medical University Luzhou City China

**Keywords:** colorectal cancer, gut microbiota, immune checkpoint inhibitors(ICIs), immunotherapy, tumor microenvironment (TME)

## Abstract

**Background:**

Colorectal cancer (CRC) is among the most prevalent malignant tumors in the digestive system and is the third leading cause of cancer‐related mortality. In recent years, immunotherapy has markedly enhanced the objective response and survival rates for CRC patients. However, the therapeutic efficacy of immunotherapy remains insufficient for the majority of proficient mismatch repair (pMMR) CRC patients, with 20% to 30% of deficient mismatch repair (dMMR) patients demonstrating poor responses or developing drug resistance. Increasing evidence underscores the critical role of intestinal microorganisms in modulating the effectiveness of immunotherapy, particularly in regulating the tumor microenvironment (TME).

**Methods:**

This review investigates the differences in intestinal microbiota and TME between dMMR and pMMR CRC. It explores how intestinal microbial communities influence TME components, including immune cells, macrophages, and fibroblasts, thereby impacting the response to immunotherapy.

**Conclusion:**

Intestinal microorganisms play a critical role in the effectiveness of immunotherapy. Variations in intestinal microbiota and the TME among patients with different mismatch repair deficiencies can significantly influence the efficacy of immune checkpoint inhibitors. Modulating the intestinal microbiota has the potential to enhance the therapeutic response of CRC to immunotherapy.

**Abbreviations**
CAFs: Cancer‐associated fibroblasts
CRC: Colorectal cancer
CTLA‐4: cytotoxic T‐lymphocyte‐associated protein 4
DCs: dendritic cells
dMMR: mismatch repair deficiency
ECM: extracellular matrix

*F. nucleatum*

:
*Fusobacterium nucleatum*

FMT: Fecal microbiota transplantation
ICIs: immune checkpoint inhibitors
IFN‐γ: interferon‐gamma
IL‐12: interleukin‐12
IL‐17: interleukin‐17
IL‐23: interleukin‐23
LAG‐3: lymphocyte activation gene 3
MDSCs: myeloid‐derived suppressor cells
MSI: Microsatellite instability
MSI‐H: microsatellite instability‐high
MSI‐L: microsatellite instability‐low
MSS: microsatellite stability
NK: natural killer
ORR: objective response rate
PD‐1: programmed cell death protein 1
PD‐L1: programmed cell death ligand 1
pMMR: proficient mismatch repair
ROS: reactive oxygen species
SCFAs: short‐chain fatty acids
TAMs: Tumor‐associated macrophages
TCM: Traditional Chinese Medicine
TGF‐β: transforming growth factor‐beta
Th1: T helper 1
TIGIT: T cell immunoreceptor with Ig and ITIM domains
TILs: tumor‐infiltrating lymphocytes
TIME: Tumor Inflammatory Microenvironment
TME: tumor microenvironment
TNF: tumor necrosis factor
Treg: regulatory T cells
VEGF: Vascular endothelial growth factor

## 1 Introduction

Colorectal cancer (CRC) is one of the most prevalent malignancies affecting the digestive system, ranking as the second most frequently diagnosed cancer and the third leading cause of cancer‐related mortality worldwide [1]. According to data from the American Cancer Society, the incidence rates of CRC have been increasing since 2011, with both regional and distant metastatic cases rising by approximately 3% annually [1, 2]. Projections indicate that by 2030, the global incidence of CRC will increase by 60%. Despite significant advancements in conventional treatments, including surgery, chemotherapy, and radiotherapy, the overall survival rates for CRC patients remain discouraging, particularly in cases of advanced disease or distant metastasis, with the current five‐year survival rate being approximately 12.5% [3].

In this context, immunotherapy is emerging as a promising treatment modality for CRC. It enhances the capacity of immune cells to recognize and attack cancer cells by activating the body's immune system. Common immune checkpoint inhibitors (ICIs) include cytotoxic T‐lymphocyte‐associated protein 4 (CTLA‐4) inhibitors, such as ipilimumab; programmed cell death protein 1 (PD‐1) inhibitors, like nivolumab; and programmed cell death ligand 1(PD‐L1) inhibitors, such as atezolizumab. These agents inhibit the function of checkpoint proteins, thereby improving the ability of immune cells to target tumor cells [4]. Clinical studies have demonstrated that the objective response rate (ORR) for CRC patients receiving immunotherapy ranges from 30% to 60%, while the one‐year overall survival rate varies between 72% and 85% [5].

Current clinical research demonstrates that immunotherapy is particularly effective in CRC patients with microsatellite instability‐high (MSI‐H) or mismatch repair deficiency (dMMR) [6, 7]. These patients typically present with an immunologically “hot” tumor microenvironment (TME), characterized by a significantly increased tumor mutational load. This condition promotes the functional remodeling of cytotoxic CD8+ T cells and CD4+ T cells, which further activates the TME and enhances the response to ICIs [8, 9]. However, despite ongoing immunotherapy, approximately 20%–30% of dMMR CRC patients still experience poor efficacy or develop resistance to treatment [10]. In contrast, patients with proficient mismatch repair (pMMR) CRC typically exhibit an immunologically “cold” TME, marked by insufficient immune cell populations, resulting in a poor or even ineffective response to immunotherapy.

An increasing body of research highlights the crucial role of gut microbiota and their metabolites in modulating the effects of immunotherapy, particularly concerning the composition of the TME. These microorganisms influence various components, including immune cells, macrophages, and fibroblasts within the TME, significantly impacting the efficacy of immunotherapy [11, 12]. Furthermore, differences in the gut microbiota between patients with dMMR and pMMR tumors suggest that intestinal microorganisms may be a key factor influencing the response to immunotherapy [13]. In light of these findings, this review focuses on the differing responses of dMMR and pMMR patients to immunotherapy, investigates the distinctions in their TME characteristics and gut microbiota composition, and examines how the gut microbiota may modulate the efficacy of immunotherapy by altering the TME. Ultimately, it aims to provide new strategies for enhancing the effectiveness of immunotherapy.

## 2 The TME of dMMR and pMMR CRC: The Contrast Between Cold and Fever

Microsatellite instability (MSI) refers to mutations in the microsatellite regions of DNA, typically resulting from failures in DNA repair mechanisms. In addition to dMMR, factors such as DNA hypermethylation and epigenetic mutations also contribute to MSI. Based on the degree of variation in the microsatellite regions in CRC, MSI is commonly categorized into three subtypes: MSI‐H, microsatellite instability‐low (MSI‐L), and microsatellite stability (MSS) [14]. Studies indicate that approximately 85%–90% of CRC patients exhibit the pMMR type, while around 10%–15% display the dMMR type of MSI [15]. For the purpose of group presentation and analysis, this review, like most in the field, classifies patients into MSS/pMMR and MSI‐H/dMMR groups. MSI‐L, which lies between MSI‐H and MSS, shares clinical and biological characteristics more similar to MSS and is therefore typically classified within the pMMR group [16].

The TME is a complex biological system that includes tumor cells and various other cell types, such as immune cells, fibroblasts, macrophages, vascular endothelial cells, regulatory T cells (Treg), and myeloid‐derived suppressor cells (MDSCs), along with components of the extracellular matrix (ECM) [17]. These cells and components interact to promote tumor growth, invasion, and metastasis. Inflammation plays a crucial role in the TME, influencing immune responses and inflammatory factors, and further modulating its function and characteristics [18]. dMMR CRC is often characterized by significant immune cell infiltration and an active immune response, resulting in a “hot” TME that facilitates the immune system's recognition and attack on tumor cells. In contrast, pMMR CRC typically exhibits minimal immune cell infiltration and presents a “cold” TME, which is closely associated with immune escape mechanisms and an inability to effectively engage in tumor immune surveillance.

### 2.1 T Cells and NK Cells

The interaction between the status of CRC and its microenvironment modulates the host immune response by influencing the phenotype and function of immune cells. Due to deficiencies in the mismatch repair system, dMMR CRC tumor cells express a greater variety of heterogeneous antigens on their surface, which attracts increased infiltration of CD8+ T cells [19]. This infiltration facilitates the identification and destruction of tumor cells expressing these heterogeneous antigens. Elevated levels of CD8+ T cell infiltration are generally associated with improved patient outcomes in response to immunotherapy [20]. Further research indicates that CRC cells within an active T helper 1 (Th1)/cytotoxic T lymphocyte (CTL) immune microenvironment tend to express high levels of various immunosuppressive‐related molecules, including PD‐1, CTLA‐4, lymphocyte activation gene 3 (LAG‐3), and T cell immunoreceptor with Ig and ITIM domains (TIGIT) [21]. This expression results partly from active immunity, prompting tumor cells to adapt and evade immune clearance by upregulating these immunosuppressive molecules. Simultaneously, to maintain homeostasis in response to a robust immune challenge, the host organism upregulates these immunosuppressive molecules through a negative feedback mechanism [22]. In vitro studies demonstrate that the cytokine interferon‐gamma (IFN‐γ), produced by Th1 cells and natural killer (NK) cells, plays a crucial role in this immune response. It promotes the production of various immunosuppressive ligands by activating the JAK–STAT signaling pathway, thereby enhancing the activation of effector T cells [23].

pMMR CRC is classified as an “immune cold” tumor due to its lower mutation burden and insufficient generation of diverse neoantigens necessary to provoke T cell responses, resulting in reduced immune cell infiltration [24]. Research indicates that the immune microenvironment of this CRC subtype contains a limited number of effector T cells, with over 60% of NK cells expressing PD‐1. This expression facilitates binding to malignant cells, leading to the release of degranulating cytokines, which ultimately results in NK cell dysfunction and compromises their immune surveillance capabilities [25]. Consequently, the low mutation burden and the development of an immunosuppressive TME in pMMR CRC contribute to a consistently low level of immune cell infiltration, correlating with a poor or even ineffective response to ICIs.

### 2.2 Tumor‐Associated Macrophages

Tumor‐associated macrophages (TAMs) can be classified into M1 (pro‐inflammatory) and M2 (anti‐inflammatory) subtypes. M1 macrophages are typically activated by IFN‐γ or tumor necrosis factor (TNF) and are capable of producing nitric oxide and reactive oxygen species (ROS), which exert cytotoxic effects on cancer cells [26, 27]. Sumana and colleagues found that patients with dMMR CRC generally exhibit a higher proportion of M1 macrophages due to increased immune cell infiltration [28]. Furthermore, M1 macrophages activate and enhance the antitumor activity of CD8+ T cells and NK cells by secreting pro‐inflammatory factors and chemokines, such as CCL5 and CXCL9, thereby further enhancing the immune microenvironment in dMMR patients [29]. In contrast, patients with pMMR CRC display higher levels of M2 macrophages, which are more likely to promote tumor growth and inhibit T cell activity [30]. Fortunately, this polarized state of macrophages is not permanent and can be modulated to switch between M1 and M2 phenotypes through interventional regulation.

### 2.3 T Regulatory Cells and Myeloid‐Derived Suppressor Cells

Some studies report significant differences in the numbers of Tregs and MDSCs between pMMR and dMMR CRC, while other studies find no significant differences [31, 32]. However, assessing the quantity and function of these cells necessitates a comprehensive analysis of the TME. dMMR CRC is associated with increased immune cell infiltration, which may result in a relatively lower presence of Tregs and MDSCs, whereas pMMR CRC exhibits pronounced immunosuppression. In this microenvironment, the number of Tregs and MDSCs is comparatively higher, and their functions are more active [21, 31]. These cells inhibit the activity of T cells and antigen‐presenting cells by producing inhibitory cytokines such as interleukin‐17 (IL‐17) and transforming growth factor‐beta (TGF‐β), as well as immunosuppressive molecules such as CTLA‐4. This immunosuppressive environment facilitates tumor evasion from immune surveillance, with Tregs and MDSCs further enhancing the immunosuppressive TME by promoting each other's functions [33, 34]. Additionally, research categorizes Tregs into activated and non‐suppressive subtypes, with tumor‐infiltrating Tregs predominantly comprising suppressive subtypes that are strongly correlated with resistance to ICIs and poor prognosis in CRC patients [35].

### 2.4 Endothelial Cells

Endothelial cells are influenced by various angiogenic factors that not only promote the proliferation of tumor blood vessels but also collaboratively shape the immunosuppressive TME by modulating immune cell activity [36]. Vascular endothelial growth factor (VEGF) is a key regulator of angiogenesis. Research demonstrates that the overexpression of VEGF enhances the expansion of Tregs and MDSCs, leading to hypoxia and elevated lactate levels within the TME. In this context, the expression of immune checkpoints such as PD‐1, CTLA‐4, TIM3, and LAG3 also significantly increases [37, 38]. Furthermore, VEGF can indirectly impair the differentiation of precursor cells into CD4+ and CD8+ T cells by affecting T cell activity and the local immunosuppressive environment, while also diminishing the antigen‐presenting capacity of dendritic cells (DCs) [39]. The Notch signaling pathway regulates the expression of endothelial cells and angiogenic factors like VEGF through the interaction of Notch receptors with ligands such as Delta‐like ligand 4, influencing the differentiation of T cells into various subtypes [40]. Additional studies indicate that Notch signaling facilitates the polarization of TAMs toward immunosuppressive M2 macrophages while simultaneously enhancing the expression of PD‐L1 [41].

dMMR CRC typically demonstrates reduced angiogenic activity. This trait may be linked to the high density of immune cells, an active immune response, and elevated expression of cytokines in dMMR tumors. For example, studies show that M1 macrophages can secrete anti‐angiogenic factors such as interleukin‐12 (IL‐12) and interleukin‐23 (IL‐23), which inhibit the formation of tumor blood vessels [42, 43]. Furthermore, IFN‐γ can suppress the expression of the angiogenic factor VEGF and induce apoptosis in endothelial cells [44]. In contrast, pMMR CRC is characterized by an enrichment of the Notch pathway, which contributes to the development of an immunosuppressive TME that enhances tumor angiogenic activity [43].

### 2.5 Cancer‐Associated Fibroblasts

Cancer‐associated fibroblasts (CAFs) exhibit functions akin to those of mesenchymal stem cells by facilitating the differentiation of Treg cells through the secretion of cytokines and chemokines, including TGF‐β and platelet‐derived growth factor [45]. Concurrently, CAFs inhibit the activation of CD8+ T cells, thereby preserving the tumor's immunosuppressive milieu. Furthermore, CAFs play a critical role in tumor vascularization by remodeling the ECM [46]. In patients with dMMR CRC, the functional capacity of CAFs may be constrained due to the elevated immunogenicity of the tumor. Research conducted by Dan Zhang and colleagues demonstrates that in dMMR CRC, CD8+ T cells can infiltrate both normal mucosa and cancer stroma to a certain extent, even in the absence of CAFs [47]. Additionally, studies in murine models indicate that mutations in the TGF‐β receptor type 2 can induce MSI in CRC [48]. Clinical investigations further confirm that mutations in receptor type 2 are associated with an improved prognosis in dMMR CRC patients [49]. Notably, the upregulation of TGF‐β and its associated substrates, secreted and converted by CAFs, contributes to drug resistance and poor prognosis in dMMR CRC patients [45, 49]. In contrast, in patients with pMMR CRC, CAFs do not exhibit functional inhibition. In this context, TGF‐β secreted by CAFs further suppresses the differentiation of Th1 cells and the activity of cytotoxic T cells within the TME, promotes the generation of Tregs, and fosters an “immune cold” microenvironment [29, 50].

### 2.6 Tumor Inflammatory Microenvironment

Tumor cells recruit immune cells by secreting inflammatory mediators, which subsequently release additional inflammatory factors, thereby creating a self‐sustaining tumor inflammatory microenvironment (TIME) [51]. Given that the TIME is often pronounced in patients with dMMR CRC, this discussion specifically focuses on the characteristics of dMMR CRC [52]. The insights gained from this analysis may also contribute to enhancing the immunosuppressive TME in pMMR CRC.

The TIME functions as a double‐edged sword in the context of ICIs treatment. On one hand, this microenvironment facilitates immune cell infiltration into tumors by increasing the overall number of immune cells [53]. Concurrently, inflammatory signals may elevate the expression of immune checkpoint molecules, enabling ICIs to more effectively recognize and block these molecules, thereby enhancing T cell attacks on tumors [54]. Furthermore, inflammation resulting from immune cell activation and the release of pro‐inflammatory cytokines can induce DNA damage and mutations within tumors, thereby increasing their immunogenicity and attracting additional immune cells [55]. Tseng‐Rogenski et al. report that IL‐6 inflammatory cytokines disrupt the nuclear localization and DNA repair function of the MMR protein hMSH3 through signaling pathways, promoting tumor heterogeneity [56]. Additionally, under inflammatory conditions, the selective deletion of the MMR protein MLH1 in colonic epithelial cells may lead to the loss of MutL α‐specific regulation of exonuclease 1 during DNA repair. This impairment compromises the integrity of the MMR system, increases the risk of gene mutations, and results in a reactive immune heat TME in dMMR CRC patients [57].

**FIGURE 1:**
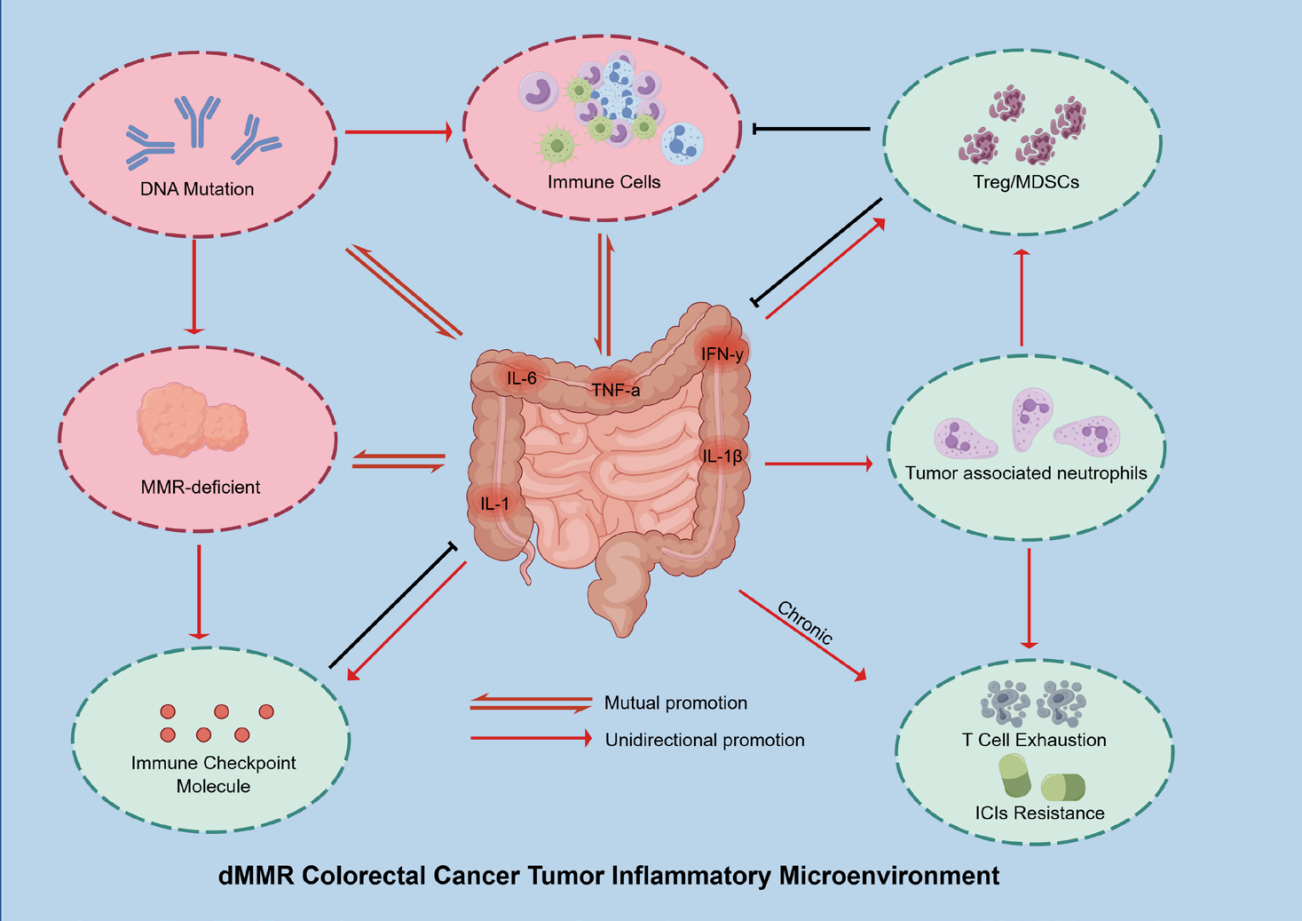
TIME mechanisms of dMMR CRC:Tumor cells that are rich in neoantigens and exhibit high levels of immune cell activity secrete or accumulate inflammatory factors and mediators, thereby increasing inflammation within the TME. Concurrently, these elevated inflammatory factors and mediators promote the development of the dMMR state and enhance immune cell activity by inducing tumor DNA damage and mutations, as well as mediating inflammatory responses. However, these inflammatory factors and mediators also partially limit the excessive accumulation of immune cells by upregulating immunosuppressive cells and immune checkpoint molecules. Furthermore, inflammatory factors associated with chronic inflammation contribute to persistent T cell exhaustion, ultimately leading to resistance to ICIs. ICIs resistance, Immune Checkpoint Inhibitors Resistance; IFN‐γ, Interferon Gamma; IL‐1, Interleukin 1; IL‐1B, Interleukin 1 Beta; IL‐6, Interleukin 6; MDSCs, Myeloid‐Derived Suppressor Cells; MMR‐deficient, Mismatch Repair‐Deficient; Treg, Regulatory T cells; TNF‐α, Tumor Necrosis Factor Alpha.

In contrast, the increase in pro‐inflammatory cytokines within the TIME establishes a feedback mechanism that promotes the expansion of immunosuppressive cell subpopulations [58]. Specifically, tumor‐associated N2 neutrophils facilitate tumor growth and angiogenesis by secreting cytokines such as ARG2 and VEGF. They also promote the accumulation of Tregs and enhance the production of other immunosuppressive factors, thereby creating an immunosuppressive TME [59, 60]. Furthermore, prolonged inflammatory responses and the overexpression of immune checkpoint molecules lead to T cell exhaustion, complicating the resumption of immune attacks on tumors [61]. Recent single‐cell sequencing studies have also indicated that IL‐1β is closely associated with resistance to anti‐PD‐1 therapy [62] (shown in Figure 1).

## 3 The Effect of Gut Microbiota on TME

**FIGURE 2:**
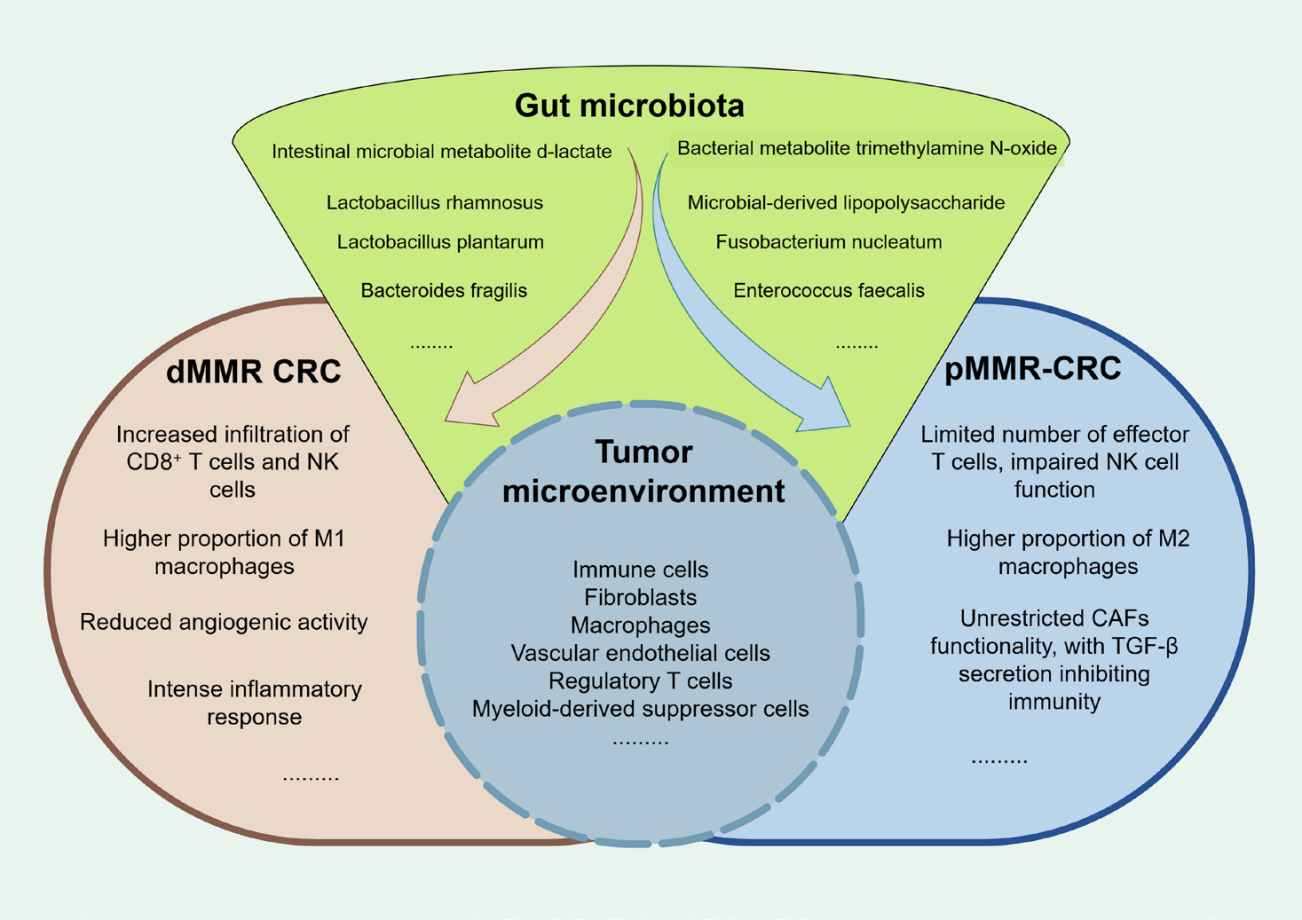
Gut Microbiota Promotes the Adaptive Transformation of the TME in CRC: dMMR CRC typically exhibits a TME characterized by higher levels of CD8^+^ T cell and NK cell infiltration due to its greater immunogenicity, along with lower levels of Tregs and MDSCs, and a higher proportion of M1 macrophages. In contrast, pMMR type CRC presents an opposite scenario. Furthermore, the gut microbiota induces changes in the TME by promoting cell proliferation and enhancing cellular activity. For instance, *Bacteroides fragilis* stimulates the proliferation and differentiation of natural killer T cells in mice, leading to the production of the immunoregulatory cytokine IL‐10, thereby boosting immune cell activity. This process makes the TME more inclined toward dMMR‐type alterations, ultimately enhancing the efficacy of immunotherapy.

In addition to the immune cell composition and tumor cell characteristics of the TME, an increasing body of research indicates that the gut microbiota plays a crucial role in shaping the TME [63]. The status, diversity, and metabolites of the gut microbiota can interact directly or indirectly with tumor cells, influencing their proliferation and apoptosis, as well as modulating the host's immune response and inflammatory state. This interaction promotes the adaptive transformation of the TME [64]. Therefore, as a mediator of TME regulation, the modulation of the intestinal microbiota can produce synergistic effects on the TME and enhance the efficacy of immunotherapy (as illustrated in Figure 2).

### 3.1 Modulate Immune Cell Function

Gut Microbiota and their metabolites can influence immune cell activity through both direct and indirect mechanisms. Garth Cameron and colleagues demonstrated that *Bacteroides fragilis* induces the proliferation and differentiation of mouse NKT cells into the immune regulatory cytokine IL‐10, thereby enhancing immune cell activity [65]. Concurrently, bacterial immune stimulation facilitates the formation of memory T cells, which are essential for sustaining long‐term antitumor immunity and reducing the likelihood of tumor recurrence [66]. Ying Zhang et al. found that the SAMphIF and SAMpmIF strains, derived from attenuated strains of *Salmonella typhimurium* , effectively inhibited tumor growth in mice with MC38 and CT26 subcutaneous tumors, as well as enhanced proliferation and activation of CD4^+^ T, CD8^+^ T, and NK cells within tumor tissue. Also, after these bacteria eradicated tumors, ≥ 50% of the mice showed no evidence of tumor recurrence after rechallenge with the same tumor cells [67]. In contrast, Erik Thiele Orberg et al. found that the combined effect of fragile Bacteroidetes enterotoxin BFT and IL‐17 on colonic epithelial cells promotes the differentiation of tumor monocytes MDSCs, selectively upregulates Arg1 and Nos2, produces NO, and inhibits T cell proliferation [68].

### 3.2 Manage the Homeostasis of Immunosuppressive Cells

Bacterial strains and their metabolites can also regulate the balance of immunosuppressive cells. Sang‐Kap Han et al. demonstrated through mouse studies that the oral administration of *Lactobacillus rhamnosus* HDB1258 significantly enhances the activity of NK cells and macrophages in the intestine while inhibiting the differentiation of Th1 and Treg cells [69]. Concurrently, deaminotyrosine, a metabolite derived from Bacteroides and Clostridium, was found to enhance the positive feedback loop of IFNAR1 through STAT1, thereby improving T cell priming and IFN‐I signaling while inhibiting the activity of immunosuppressive cells [70]. On the contrary, when *Fusobacterium nucleatum* ( *F. nucleatum* ) was administered orally to mice, the levels of pro‐inflammatory cytokines—such as IL‐6, IL‐12, IL‐9, IL‐17A, CXCL1, TNF‐α, and IFN‐γ—were significantly increased in the mouse plasma. Simultaneously, the liver immune response contributes to CRC liver metastasis by reducing immune cell activity, recruiting MDSCs, and increasing the accumulation of Treg cells in the liver [71].

### 3.3 Steer Macrophage Polarization

The status of gut microbiota significantly influences the polarization of macrophages within the TME. The study conducted by Ting Chen et al. demonstrated that macrophages are the predominant type of tumor‐infiltrating immune cells in CRC infected by *F. nucleatum* . Utilizing the ApcMin/+ mouse model, the research revealed that *F. nucleatum* infection promotes CRC growth through a TLR4‐dependent mechanism, activates the IL‐6/p‐STAT3/c‐MYC signaling pathway, and induces M2 polarization of macrophages, thereby exerting an immunosuppressive effect [72]. In a murine model of tumorigenesis, Rui Li et al. found that microbial‐derived lipopolysaccharide/metastasis‐related secretory protein cathepsin K mediates the chemokine‐dependent recruitment of monocyte‐like macrophages, a process that enhances intestinal permeability and allows for the excessive release of microbial products, promoting M2‐like macrophage differentiation and creating an invasion– metastasis microenvironment [73]. Additionally, as an endogenous immune modulator, the intestinal microbial metabolite d‐lactate can reprogram M2 macrophages into M1 macrophages and reshape the TME in mice by inhibiting the PI3K/Akt pathway [74].

### 3.4 Regulates Tumor Angiogenic Signaling

Gut microbiota can selectively activate mucosal endothelial and mesenchymal cells to promote specific angiogenic responses in a manner dependent on TLRs and NOD‐like receptors [36]. Juanjiang Chen et al. demonstrated that the administration of *Lactobacillus plantarum* WLPL09 induces tumor cell apoptosis in B16F10 melanoma tumor‐bearing mice, downregulates the angiogenesis markers VEGF and FGF2, and inhibits tumor angiogenesis [75]. Similarly, *Clostridium butyricum* reduces tumor angiogenesis by downregulating the expression of METTL3 and decreasing the levels of vimentin and vascular endothelial growth factor receptor 2 in CRC cells [76]. In contrast, the bacterial metabolite trimethylamine N‐oxide upregulates angiogenic factors such as VEGF, significantly promoting CRC tumor proliferation and angiogenesis. This mechanism may be associated with migration‐related signaling pathways, including the PI3K/Akt and MAPK pathways, as well as an increase in oxidative stress levels [77].

### 3.5 Control the Inflammatory Cascade

An imbalance in bacterial flora can lead to damage of the intestinal barrier, facilitating the penetration of bacteria or their endotoxins, which subsequently triggers inflammation [78, 79]. The inhibition of autophagy in CRC epithelial cells mediated by *F. nucleatum* can promote the accumulation of ROS and induce the production of pro‐inflammatory cytokines, such as IL‐8, IL‐1β, and TNF‐α [80]. Furthermore, Enterotoxigenic *Bacteroides fragilis* activates a STAT3‐NF‐κB‐dependent pro‐inflammatory signaling cascade, resulting in the release of cytokines like IL‐17 and IL‐23, which have been shown to attract proneoplastic myeloid cells and promote distal colon tumorigenesis [81]. In contrast, Ruiyue Sun et al. demonstrated that *Bacillus subtilis* JLCC513 enhanced the abundance of lactobacilli and reduced the levels of Helicobacter spirochetes, thereby decreasing intestinal inflammation in mice through the inhibition of the TLR4/NF‐κB/NLRP3 pathway [82].

## 4 The Role of Gut Microbiota in dMMR/pMMR CRC

### 4.1 Differences and Roles of Gut Microbiota in dMMR/pMMR CRC

**FIGURE 3:**
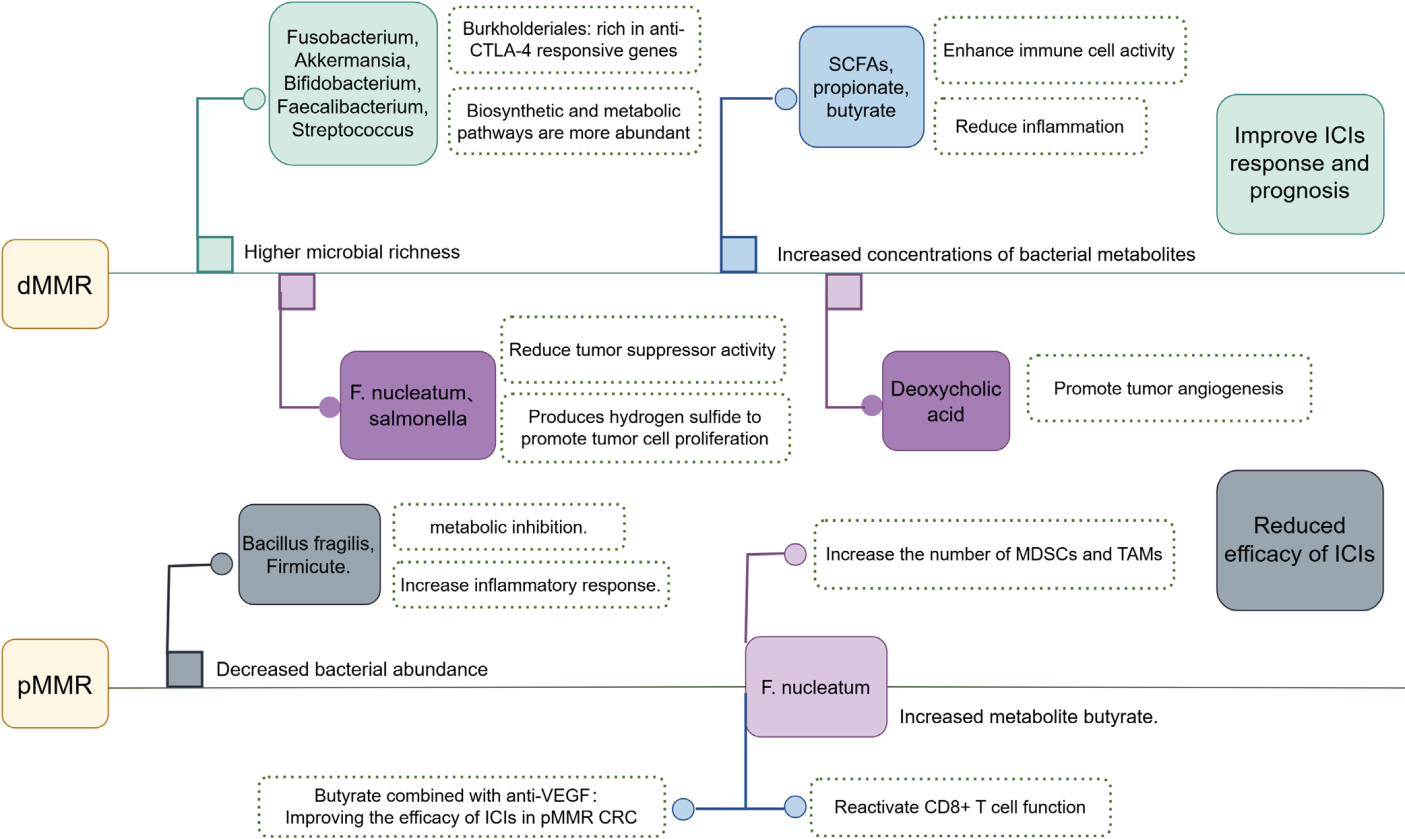
Differences in gut microbiota in dMMR/pMMR CRC:Compared to patients with proficient mismatch repair (pMMR), those with deficient mismatch repair (dMMR) colorectal cancer (CRC) exhibit greater diversity and abundance of gut microbiota, including a higher presence of probiotic genera. This is accompanied by an increase in bacterial metabolites and associated metabolic pathways, which contribute to enhanced immune cell activity and improve the efficacy of immune checkpoint inhibitors (ICIs). In contrast, pMMR patients demonstrate lower bacterial abundance, which diminishes the positive regulatory effects on immune cells and the tumor microenvironment (TME). However, certain controversial ‘harmful bacteria’ (e.g., *Fusobacterium nucleatum* ) may still enhance the immunotherapy response in pMMR patients to some extent. Anti‐CTLA‐4, Anti‐Cytotoxic T‐Lymphocyte Antigen 4; Anti‐VEGF, Anti‐Vascular Endothelial Growth Factor; *F. nucleatum, Fusobacterium nucleatum* ; ICIs, Immune Checkpoint Inhibitors; MDSCs, Myeloid‐Derived Suppressor Cells; pMMR CRC, Proficient Mismatch Repair Colorectal Cancer; SCFAs, Short‐Chain Fatty Acids; TMAs, Tumor‐associated macrophages.

In a study involving 230 patients, Min Jin et al. found that dMMR CRC exhibits higher microbial richness and distinct compositional differences compared to pMMR CRC. Notably, at the genus level, patients with dMMR CRC show a significant presence of Fusobacterium, Akkermansia, Bifidobacterium, Faecalibacterium, Streptococcus, and other species. KEGG pathway analysis reveals that, compared to the pMMR group, the biosynthesis and metabolism pathways of glycans and nucleotides, as well as pathways related to cell growth, death, gene replication, and repair, are more abundant in the dMMR group [83]. Furthermore, machine learning models used to predict treatment responses to ICIs indicate that a higher abundance of gut microbiota, such as Fusobacterium and Faecalibacterium, correlates with improved immunotherapy responses and prognosis [84]. In microbial metabolomics research, an increase in the abundance of probiotics such as Faecalibacterium, Bacteroides, and Fibrobacter correlates with a rise in the concentration of bacterial metabolites, including propionate and butyrate, in the intestine. These metabolites can further enhance immune cell activity and infiltration [85]. Additionally, the relative abundance of Bacteroidales and Burkholderiales is significantly higher in the gut microbiota of the mouse dMMR CRC model, with Burkholderiales being the only bacterial category enriched in anti‐CTLA‐4 responders [86] (Figure 3).

Recent research indicates that short‐chain fatty acids (SCFAs) are significantly enriched in dMMR CRC and induce DNA damage in CRC cells by upregulating major histocompatibility complex class I and chemokines. This mechanism activates cytotoxic CD8 T cells and DCs, thereby enhancing the efficacy of ICIs [87, 88, 89]. Furthermore, Hamada et al. report that patients with dMMR CRC exhibit a higher burden of *F. nucleatum* in the gut, often correlating with lower levels of T cell infiltration and poorer clinical prognostic outcomes [90]. Additional studies demonstrate that the enrichment of *F. nucleatum* significantly correlates with TAM infiltration and CDKN2A (p16) promoter methylation in dMMR CRC patients. This modification contributes to tumor cell proliferation and development by diminishing tumor suppressor activity [91]. Concurrently, dMMR CRC shows an increased capacity for hydrogen sulfide production due to the enrichment of Salmonella. The elevated hydrogen sulfide levels may promote tumor cell survival and proliferation, potentially impacting the efficacy of immunotherapy in patients [92, 93].

*F. nucleatum* , often recognized as a pathogen in the progression of CRC, is also present in patients with pMMR CRC. It negatively impacts tumor‐specific immune responses by increasing the number of MDSCs and TAMs within the TME, thereby reducing the efficacy of ICIs therapy [91, 94]. Notably, some studies have demonstrated that *F. nucleatum* possesses unique advantages in enhancing the efficacy of ICIs. Research conducted by Yaohui Gao et al. indicates that elevated levels of *F. nucleatum* are associated with improved therapeutic responses to PD‐1 blockade in CRC patients [95]. Furthermore, Xueliang Wang et al. discovered that *F. nucleatum* suppresses the expression of PD‐1 in CD8+ tumor‐infiltrating lymphocytes (TILs) through its metabolite butyrate in the CRC TME. This suppression occurs via the inhibition of histone deacetylases HDAC3 and HDAC8, leading to an increase in TBX21 expression, which subsequently reactivates the antitumor function of CD8+ T cells in MSS mice [96]. This seemingly contradictory finding highlights the potential of *F. nucleatum* in tumor therapy. One contributing factor may be the varying pathogenicity among different subspecies of *F. nucleatum* , including nucleatum, animalis, vincentii, and polymorphum [97]. However, a more significant consideration is the regulatory effect of *F. nucleatum* on the TME. Existing studies indicate that *F. nucleatum* can enhance the efficacy of pMMR CRC immunotherapy, primarily due to its ability to simultaneously boost the activity of immune cells, such as CD8+ T cells and CD34+ cells [95, 97, 98]. Furthermore, *F. nucleatum* exhibits notable pro‐inflammatory effects by activating the TLR4/MYD88/NF‐κB signaling pathways or the Wnt/β‐catenin pathways [99, 100]. Consequently, *F. nucleatum* may serve as a potential adjuvant to improve the efficacy of ICIs by promoting antigen immunity, thereby shifting the TME toward a “hot” state. Nevertheless, it is crucial to remain cautious of its potential risks in promoting cancer. Future treatment strategies may consider introducing probiotics to counteract *F. nucleatum* , while also employing microbial community therapy to comprehensively activate antitumor immune responses.

### 4.2 Regulation of CRC Immunotherapy

**FIGURE 4:**
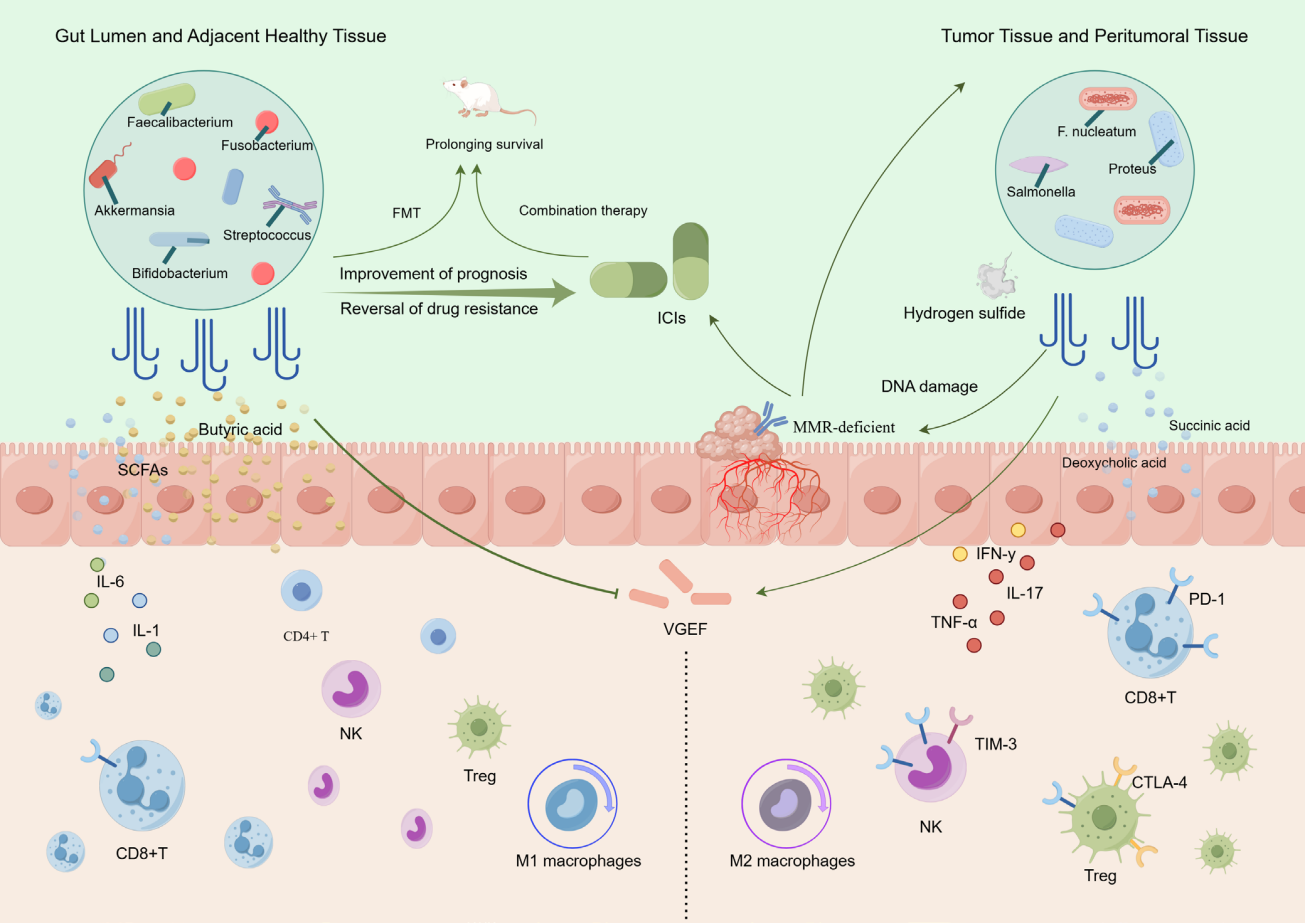
Gut microbiota improves the efficacy of ICIs by regulating TME: The enrichment of beneficial bacteria, such as Akkermansia and Bifidobacterium, enhances the efficacy of ICIs. The metabolites produced by these beneficial bacteria, including SCFAs and acetic acid, inhibit the activity of immunosuppressive cells and tumor angiogenesis. Furthermore, they activate immune cell functions, promote the polarization of M1 macrophages, and regulate the secretion of anti‐inflammatory cytokines. The administration of oral probiotics or gut microbiota transplantation has been shown to improve ICI efficacy and extend the median survival of experimental mice. Conversely, the enrichment of harmful bacteria, such as Proteus and *F. nucleatum* , contributes to the development of an immunosuppressive TME by promoting the generation of immunosuppressive cells and checkpoint molecules, polarizing M2 macrophages, and inhibiting immune cell activity. Notably, certain harmful bacteria, including *Escherichia coli* and Clostridium, can alter the MMR system phenotype of tumors, thereby enhancing the response to ICIs through the induction of DNA damage and the exacerbation of inflammatory responses. CTLA‐4, Cytotoxic T‐Lymphocyte Antigen 4; FMT, Fecal microbiota transplantation; *F. nucleatum* , *Fusobacterium nucleatum* ; IFN‐γ, Interferon gamma; ICIs, Immune checkpoint inhibitors; IL‐1, Interleukin 1; IL‐6, Interleukin 6; IL‐17, Interleukin 17; MMR‐deficient, Mismatch Repair‐deficient; NK, Natural Killer cell; PD‐1, Programmed Cell Death Protein 1; SCFAs, Short‐chain fatty acids; Treg, Regulatory T cell; TNF‐α, Tumor Necrosis Factor alpha; VEGF, Vascular Endothelial Growth Factor.

The gut microbiota influences the host's immune response through various TME‐related mechanisms, which are crucial for enhancing immune cell activity in pMMR CRC. For instance, *Lactobacillus rhamnosus* Probio‐M9 promotes the infiltration and activation of CTLs, inhibits Treg function, and enhances the anti‐PD‐1 tumor‐suppressive effect by increasing the abundance of Lactobacillus, *Bifidobacterium animalis* , and pyridoxine [101, 102]. Additionally, oral administration of *Bifidobacterium breve* lw01 stimulates the upregulation of the dendritic cell‐associated chemokine CCL20, leading to the recruitment of more DCs and TILs in the intestinal villi, as demonstrated in an orthotopic ligated intestinal loop model [103]. This approach represents a method to convert “cold” tumors into “hot” tumors in patients with pMMR CRC (Figure 4). Similarly, the gut microbiota also prevents the development of resistance to ICIs in dMMR CRC by improving chronic inflammation. In the MC38 cell line, combined treatment with an anti‐PD‐L1 monoclonal antibody and attenuated Salmonella significantly enhances the therapeutic efficacy of ICIs in CRC. This improvement may be associated with a reduction in the percentage of tumor‐associated granulocytes and an increase in the infiltration of effector T cells within the tumor. Furthermore, this combination therapy may alleviate T cell exhaustion in dMMR CRC, which is often exacerbated by tumor‐associated granulocyte infiltration [104]. Additionally, the combined application of Bifidobacterium and *Enterococcus faecalis* enhances the anti‐PD‐1 therapeutic effect, likely by improving the intestinal inflammatory environment and systemic T cell activity, thereby potentially preventing the development of resistance to ICIs in dMMR CRC patients [105]. Similarly, alterations in metabolites such as arginine and SCFAs are closely linked to the improvement of primary immunotherapy resistance [88, 106].

Gut microbiota, including *Escherichia coli* and Clostridium, can produce mutagenic substances, such as Enterotoxigenic *Bacteroides fragilis* , which are capable of causing DNA damage through the secretion of carcinogenic toxins. This process may further reprogram the “cold” TME in patients with pMMR CRC by influencing the phenotype of the MMR system. Concurrently, these bacteria increase the numbers of Treg and MDSCs in the bloodstream, significantly impeding the infiltration of immune cells [68, 107]. Yali Liu et al. demonstrate through murine studies that anaerobic Pseudomonas can induce and activate the immunosuppressive functions of MDSCs by stimulating CXCL1 production, which leads to diminished therapeutic effects against PD‐1 [108]. Additionally, succinic acid produced by Fusobacterium inhibits the cyclic GMP AMP synthase–IFN‐β pathway, which reduces the levels of Th1 chemokines CCL5 and CXCL10 in tumors. This reduction limits sensitivity to PD‐1 monoclonal antibodies by restricting the transport of CD8+ T cells to the TME [109, 110]. In contrast, the combined use of *Bacteroides fragilis* enhances the therapeutic efficacy of antibodies targeting CTLA‐4, while an increase in the Prevotella genus is also associated with improved prognosis in treatments targeting the PD‐1/PD‐L1 axis [111, 112]. These findings suggest that individual bacteria, specific bacterial compositions, and bacterial metabolites within the gut microbiota could serve as potential targets for enhancing the efficacy of ICIs, (Table 1).

**TABLE 1:** Research on gut microbiota improving the efficacy of immunosuppressants by regulating the TME.

Gut microbiota/metabolites	In vivo or in vitro/tumor cell lines	Action target	Immune mechanism
**Positive modulators**			
Attenuated *Salmonella typhimurium* strain [104]	In vitro:MC38 In vivo:C57BL/6	PD‐L1、LAG‐3	Enhance CD4^+^ T cell infiltration, upregulate MHC II expression, improve the antigen presentation capability of intratumoral macrophages, and decrease PD‐1 and LAG‐3 expression.
Lactobacillus RhamnosusGG [113]	In vitro:MC38 In vivo:C57BL/6	PD‐1	Enhance beneficial bacteria while reducing harmful bacteria to promote apoptosis in CRC cells, thereby synergistically improving the efficacy of anti‐PD‐1 therapy.
F. nucleatum [96]	In vitro:HT29、CT26、MC38 In vivo:C57BL/6	PD‐1	F. nucleatum inhibits the expression of PD‐1 in CD8^+^ TILs by synthesizing butyrate and epigenetically activating the TBX21 transcription factor, thereby increasing cytotoxicity of CD8^+^ TILs and tumor cell killing.
Breve (B.breve) lw01 [103]	In vitro:CT26 In vivo:BALB/c	PD‐L1	Recruit DCs and upregulate IL‐12 to promote T cell migration to tumors, thereby synergistically enhancing the efficacy of anti‐PD‐L1 therapy.
*Lactobacillus delbrueckii* subsp.bulgaricus [114]	In vitro:Colon26	CTLA‐4	Bulgaricus enhances response to ICB therapy by increasing the number of CCR6 + CD8 + T cells in lymph nodes, which can then migrate from the intestine to tumors expressing CCL20 to enhance antitumor activity
*Lactobacillus rhamnosus* Probio‐M9 [101]	In vitro:CT26 In vivo:BALB/c	PD‐1/PD‐L	Enriching a diverse array of resident symbiotic intestinal microorganisms, SCFAs, and butyric acid contributes to the accumulation of blood‐borne α‐ketoglutarate, thereby enhancing the immune‐mediated killing effect of T cells.
*Roseburia intestinalis* [115]	In vivo:CT26 In vivo:C57B6/N	TIM‐1	*Roseburia intestinalis* inhibits tumor growth by inducing cytotoxic granzyme B+, IFN‐γ, and TNF‐α + CD8 + T cells, significantly enhancing the anti‐PD‐1 efficacy in mice carrying MSI low CT26 tumors.
*Bifidobacterium breve* JCM92 [102]	In vitro:MC38 In vivo:C57B6/N	PD‐1、E2F/NFκB/TNF‐α	Increased immune cell activity and IFN‐γ production were observed in mice, which triggered a robust inflammatory response mediated by TNF‐α signaling through NFκB.
Clostridium‐mixture [116]	In vitro:MC38‐GFP、CT26	PD‐1	By activating CD8^+^ T cells and enriching beneficial bacteria, significantly enhances the antitumor immune response when combined with anti‐PD‐1 therapy.
Inosine [117]	In vitro:MC38	CTLA‐4	The metabolite inosine activates antitumor T cells by upregulating DCs、IL‐12and IFN‐γ, thereby enhancing anti‐CTLA‐4‐induced antitumor immunity through costimulation.
Extracellular vesicles [118]	In vitro:MC38、SW620 In vivo:C57BL/6	PD‐1	EV modulates intestinal immunity and enhances the efficacy of anti‐PD‐1 therapy by increasing the proportion of immune cells 、MHC II molecules and beneficial bacteria within tumor tissuees.
Outer membrane vesicles [119]	In vitro:CT‐26 、Caco‐2、HT‐29‐MTX‐E1 In vivo:Balb/c	PD‐1	The combination of OMVs and anti‐PD‐1 can enhance the infiltration of CD8^+^ cytotoxic T lymphocytes while also enriching the populations of commensal bacteria.
SCFA [120]	In vitro:MSI mutated MC38 In vivo:C57B6/N	cGAS/STING	Inducing the feedback loop of IFN γ, thereby upregulating CRC MHCI expression and enhancing T cell activation, further activating the cGAS/STING signaling pathway, and causing DNA damage.
Urolithin B [121]	In vivo:C57BL/6	PD‐L1	Enhance the activity of NK cells, inhibit the function of Treg, reduce the expression of PD‐L1, and improve antigen presentation.
**Inhibitor**			
IDDF2022‐ABS‐0088 *Peptostreptococcus Anaerobius* [108]	In vitro:HCT116、Caco2、MC38	PD‐1	Stimulating the production of CXCL1 induces the function of MDSCs, inhibits T cell activation, and further diminishes the efficacy of anti‐PD‐1 treatment.
F. nucleatum (succinic acid) [109]	In vitro:MC38、CT26	cGAS‐STING‐IFN‐β、PD‐1/PD‐L1	Succinic acid reduces the levels of Th1 chemokines CCL5 and CXCL10 in tumors by inhibiting the cGAS IFN—β pathway, thereby reducing sensitivity to PD‐1 monoclonal antibodies by restricting the transport of CD8^+^T cells to TME.

## 5 Gut Microbiota Improves Diversified Strategies for Immunotherapy

Patients with CRC often experience an imbalance in their gut microbiota, which exhibits specific characteristics. This imbalance reduces the immune system's ability to recognize and eliminate tumors. Regulating the gut microbiota can help restore balance and enhance the effectiveness of ICIs by reshaping the TME. However, due to the dynamic nature and individual variability of both the gut microbiota and the TME, multiple strategies are required to achieve dynamic and diversified treatment approaches. These strategies can further improve the efficacy of ICIs in patients with pMMR/dMMR CRC [122].

### 5.1 Strategies for Supplementing or Modifying Single Bacterial Species

#### 5.1.1 Beneficial Bacteria

The oral administration of beneficial bacteria, such as *Bifidobacterium breve* JCM92 [102], Clostridium [116], *Lactobacillus rhamnosus* GG [113], and Probio‐M9 [101], has been shown to enhance immune cell activity and promote the proliferation of beneficial bacteria and metabolites, thereby synergistically improving the efficacy of ICIs. Although probiotics have demonstrated potential benefits in the treatment of CRC when used alongside ICIs, relevant clinical research remains limited, and direct clinical evidence is insufficient. Furthermore, the effects of probiotics can vary significantly among individuals, which is particularly concerning for immunocompromised patients. In these individuals, the use of probiotics may be associated with severe infections, such as bacteremia [123].

Moreover, the balance of the gut microbiota can be disrupted by factors such as exposure to broad‐spectrum antibiotics, immunosuppressants, intestinal transplantation, and other interventions, which can lead to bacterial dysbiosis, even in the absence of probiotic intake [124, 125]. In patients who do take probiotics, this disruption is observed more frequently. Molecular identification analyses have shown that probiotic isolates related to bacteremia can be found in affected patients, who are predominantly immunocompromised [126]. The entry of these isolates into the bloodstream is facilitated by intestinal barrier dysfunction, microbial overgrowth, and community imbalance, ultimately leading to bacteremia. Therefore, for patients with multiple underlying conditions or low immunity, caution is warranted when combining microbiota therapy, and close monitoring for potential signs of infection post‐intervention is essential. In patients with concurrent sepsis, several studies have suggested that regulating the microbiota can help maintain intestinal barrier integrity and reduce the severity of sepsis [127, 128, 129]. Additionally, derivatives of the gut microbiota, such as L‐valine, have been shown to protect against intestinal damage caused by sepsis and are negatively correlated with sepsis severity [130]. This highlights the dual nature of probiotics, emphasizing that their effects cannot simply be categorized as “beneficial” or “harmful.” Instead, their impact depends on factors such as microbiota diversity, usage, and specific clinical circumstances. Further research into the precise mechanisms of microbiota interaction is warranted.

#### 5.1.2 Harmful Bacteria

Common harmful bacteria, such as Enterotoxigenic *Bacteroides fragilis* [81] and Salmonella [104], are often considered causative or contributing factors in the development of CRC. However, there is a limited amount of basic research exploring the synergy between these strains and ICIs, with clinical studies being even scarcer. Interestingly, recent research has indicated that strains previously regarded as harmful, such as *F. nucleatum* [96], can enhance the efficacy of ICIs by modifying the TME. Therefore, it is essential to avoid biases against strains traditionally viewed as harmful. In fact, by mitigating the toxicity and side effects associated with these strains, we can further investigate and potentially harness their beneficial functions.

#### 5.1.3 Bioinorganic Hybrid Bacteriophage

Bacteriophages, commonly referred to as phages, are a class of viruses that specifically target bacteria. They exhibit a high degree of specificity, allowing them to target harmful bacteria while preserving beneficial gut microbiot [131]. Furthermore, phages can activate phagocytes or DCs and directly attack drug‐resistant bacteria, thereby helping to mitigate resistance to ICIs [132]. Xue Dong et al. employed phage display technology to isolate the M13 phage, which specifically binds to *Fusobacterium nucleatum* . They electrostatically assembled silver nanoparticles onto the surface capsid protein of M13 (M13@Ag). In experiments involving M13@Ag‐treated mice, it was observed that M13@Ag effectively eliminated *F. nucleatum* from the intestine and increased the abundance of butyrate‐producing bacteria. Moreover, it activated antigen‐presenting cells and reduced the population of MDSCs. When combined with ICIs (α‐PD1), this treatment significantly prolonged the overall survival of mice in an orthotopic CRC model [133].

#### 5.1.4 Nanoformulation

Nanotechnology significantly enhances drug solubility, biodistribution, and intracellular transport by encapsulating drugs within nanoparticles, thereby maximizing their local effects in the intestine and TME. Additionally, the design of nanocarriers facilitates the targeted release of specific bacteria in response to particular pH levels or enzymatic conditions in the intestine, promoting long‐term regulation and preventing systemic dysbiosis [134, 135]. Researchers have demonstrated that hybrid nanovesicles derived from *Akkermansia muciniphila* , *Bifidobacterium bifidum* , and *Bifidobacterium breve* effectively induce innate immune activation and promote dendritic cell maturation in mouse models. Studies indicate that lipopolysaccharide is abundant in situ within CRC tissue and is associated with low responsiveness to anti‐PD‐L1 monoclonal antibody (mAb) therapy. Based on this characteristic, Wantong Song et al. loaded the coding sequence of an LPS‐targeted fusion protein into a lipid‐protamine‐DNA nanoparticle system, aiming to selectively express an LPS‐capturing protein and block LPS in tumors. This nano‐capture system significantly alleviates the immunosuppressive microenvironment, promotes T‐cell infiltration into CRC tumors, and enhances the therapeutic effect of anti‐PD‐L1 mAb on CRC tumors [136]. However, there is currently a lack of clinical trials investigating the modification of individual bacteria to assist ICIs in CRC, and the associated toxicity and pharmacological metabolism require further investigation.

### 5.2 Strategies to Change Intestinal Microecology

#### 5.2.1 Fecal Microbiota Transplantation

Fecal microbiota transplantation (FMT) enhances the antitumor immune response by introducing healthy microbiota, thereby rebuilding the gut microbiota and restoring both the diversity and functionality of a patient's microbiome. FMT has demonstrated significant potential in patients with refractory CRC or those who do not respond to ICIs [137]. For instance, collecting feces from patients who respond to ICIs and administering FMT significantly improves the immunotherapy response in non‐responders [112]. Furthermore, Jiayuan Huang et al. found that FMT treatment significantly increases the abundance of beneficial bacteria in the intestinal tract of mice. This shift in microbiota also reshapes the gene function of microorganisms and the host metabolome, leading to enhanced efficacy of anti‐PD‐1 therapy [138]. A healthy microbial population boosts T‐cell activity, which synergizes with the mechanism of PD‐1 inhibitors to strengthen antitumor immune responses. Patients with pMMR CRC often experience an immunosuppressive TME, which limits their benefits from ICIs. However, combined treatment with FMT improves therapeutic outcomes for such patients, as confirmed by clinical cases [112, 139]. Additionally, FMT demonstrates significant improvements in survival rates and safety in a Phase II clinical trial that combines anti‐VEGF therapy with tislelizumab and fruquintinib for the treatment of refractory metastatic MSS CRC [140].

However, direct involvement in regulating the gut microbiota also presents higher risks beyond the previously mentioned bacteremia. These risks include unexpected immune regulatory reactions, such as loss of immune tolerance, immune suppression, and immune overreaction, as well as metabolic abnormalities, allergic reactions, and neurological effects related to the gut‐brain axis [141, 142]. Although the incidence of these risks is extremely low, their potentially serious consequences necessitate a thorough consideration of the patient's health status, immune function, and mental state when implementing microbiome modulation therapy. A deeper understanding of the mechanisms and preventive measures associated with these risks will aid in maximizing the therapeutic potential of FMT while minimizing risks as much as possible.

#### 5.2.2 Oral Mixed Probiotics and Prebiotics

Oral probiotics significantly enhance the TME and improve the efficacy of ICIs by modulating specific bacterial flora. Prebiotics, which consist of soluble fibers or sugars, also promote immune cell activity and antitumor responses by supplying essential nutrients to beneficial microorganisms [143]. Zhang et al. demonstrate that mice treated with an anti‐PD‐1 monoclonal antibody (mAb) in combination with pectin exhibit significantly reduced tumor growth compared to those receiving anti‐PD‐1 treatment alone. This effect is likely associated with enhanced T cell infiltration, activation, increased gut microbiota diversity, and a rise in butyrate‐producing bacteria [144]. Furthermore, in vivo experiments indicate that treatment with oral inulin gel increases the population of key commensal microorganisms, such as Bifidobacterium and *Enterococcus faecium* , as well as the relative abundance of their SCFAs. This treatment also enhances the recall responses of interferon‐γ and CD8 T cells, induces systemic memory T cell responses, and amplifies the antitumor activity of α‐PD1 [145]. Additionally, oral probiotics and prebiotics are well tolerated, exhibit fewer side effects, and can be flexibly combined, underscoring their potential for long‐term regulation of the gut microbiota.

#### 5.2.3 Antibiotic Treatment

In clinical trials, the use of antibiotics primarily serves as a preliminary preparation for FMT. The strategy of introducing beneficial bacteria through FMT or oral probiotics and prebiotics can enhance the immunotherapy response to ICIs. Can similar outcomes be achieved by eliminating harmful microorganisms with antibiotics?

As previously noted, treatment with metronidazole has been shown to reduce the abundance of *Fusobacterium nucleatum* in the intestine, resulting in decreased serum succinate levels and the resensitization of tumors to in vivo immunotherapy [109]. Furthermore, the ablation of harmful bacteria through antibiotic treatment facilitates immunogenic reprogramming of the TME in murine models, leading to a reduction in MDSCs and an increased expression of PD‐1 on intratumoral CD4+ and CD8+ T cells, thereby enhancing sensitivity to anti‐PD‐1 antibodies [146, 147]. Conversely, some clinical studies have indicated that post‐antibiotic treatment significantly diminishes both the diversity of the microbiome and the abundance of beneficial bacteria in the intestine, which adversely impacts the efficacy of immunotherapy in patients [148, 149]. Research involving murine models has demonstrated that tumor‐bearing mice exhibit a reduced response to PD‐1 antibody immunotherapy following the administration of broad‐spectrum antibiotics, underscoring the importance of a balanced gut microbiota for effective antitumor immune responses [150]. Therefore, antibiotic treatment that specifically targets harmful bacteria while preserving beneficial bacteria may represent a promising strategy for future therapeutic approaches.

#### 5.2.4 Bioactive Peptides

Bioactive peptides derived from the gut microbiota are small protein fragments that exhibit a wide range of biological functions. These peptides possess natural antibacterial properties and demonstrate low toxicity to host cells, which helps minimize damage to beneficial gut microbiota in CRC patients undergoing antibiotic treatment or other interventions [151]. Dhwani Haria and colleagues utilized Second Genome's discovery platform, sg4sight, to identify the microbiome‐derived peptide SG‐3‐00802. This peptide, whether administered alone or in combination with anti‐PD‐1, elicits an antitumor response and enhances overall survival in the anti‐PD‐1‐insensitive RENCA model. Moreover, when re‐challenged with RENCA cells, animals that had fully regressed tumors were able to reject newly implanted tumors, further demonstrating that the combination of SG‐3‐00802 and anti‐PD‐1 induces a durable antitumor memory response [152].

#### 5.2.5 Natural Products

Natural products exhibit a wide array of chemical structures and functional properties that can promote the growth of beneficial bacteria and modulate the immune microenvironment. Diosgenin, a natural steroidal saponin, is administered via gavage to C57BL/6 mice bearing melanoma. Following treatment, Lactobacillus and Sutterella are significantly upregulated in the intestinal tract of the mice, while Bacteroidetes are downregulated. Concurrently, there is a marked increase in CD4/CD8+ T cell infiltration and IFN‐γ expression within the tumor tissue, ultimately enhancing the therapeutic efficacy of PD‐1 antibodies by boosting T cell immunity and improving the TME [153]. Ginseng, a widely utilized herb, contains ginseng polysaccharide, which has been shown to enhance CD8+ T cell function and reduce Tregs by reshaping the intestinal microbiota and modulating tryptophan metabolism. This, in turn, contributes to the enhancement of the antitumor efficacy of αPD‐1 monoclonal antibodies [154].

#### 5.2.6 Dietary Intervention

Dietary intervention, as a relatively mild regulatory approach, offers long‐term sustainability. A study that combines dietary habits with immunotherapy (PD‐L1, CTLA‐4) finds a positive correlation between the Mediterranean diet—a widely recommended healthy eating pattern—and the response to ICIs [155]. Furthermore, it has been observed that mice on a Mediterranean diet experience a significant reduction in primary tumor volume. Additionally, research indicates that mice on a low‐fiber diet exhibit impaired cytotoxic T cell responses to anti‐PD‐1 treatment, while a high‐fiber diet promotes the proliferation of beneficial bacteria, such as bifidobacteria and lactobacilli. This, in turn, enhances the production of SCFAs, further boosting the immune functions of macrophages and dendritic cells [156, 157].

#### 5.2.7 Other Treatments

Traditional Chinese Medicine (TCM) prescriptions play a pivotal role in the TCM treatment framework and have been validated in numerous clinical trials for their ability to enhance the immunity of CRC patients, thereby improving the efficacy of immunotherapy responses. The mechanisms underlying these effects are intricately linked to the regulation of gut microbiota and the TME. For instance, Qizhen Decoction promotes the activation of effector T cells by facilitating the maturation of dendritic cells, releasing IL‐12, and activating the JAK2/STAT4 signaling pathway. Additionally, it increases the abundance of Akkermansia, which further enhances the efficacy of PD‐1 inhibitors [158].

## 6 Conclusion and Outlook

The key to current tumor immunotherapy strategies lies in enhancing T‐cell activation and overcoming immune suppression, particularly in patients with pMMR CRC [159]. Modulating the TME through the microbiome and its metabolites to mediate host immune responses is crucial for improving the immune‐suppressive TME of “cold tumors” in pMMR patients. In addition to activating immune cells by enhancing beneficial microbiota, utilizing attenuated harmful bacteria to transiently induce pro‐inflammatory responses can effectively recruit more immune cells, thereby enhancing antitumor immune activity [160, 161, 162].

In contrast, for patients with dMMR CRC, the effectiveness of immunotherapy often diminishes over time, necessitating treatment strategies aimed at sustaining efficacy and preventing the development of resistance. Research has shown that dMMR gastrointestinal cancer patients who develop resistance to immunotherapy exhibit distinct compositional characteristics in their gut microbiota. Specific microbes, such as Bifidobacterium and *B. breve* , may contribute to the onset of resistance by modulating immune responses or influencing gut metabolites [88]. Therefore, regular monitoring of changes in the gut microbiota, in conjunction with the patient's immune status, can provide valuable insights for predicting immunotherapy outcomes, adjusting treatment strategies, and facilitating personalized management [163, 164, 165].

Additionally, comparison of tumor microbiomes in patients with dMMR and pMMR CRC revealed that dMMR tumors are significantly enriched with bacteria such as *F. nucleatum* , *Bacteroides fragilis* , and *Prevotella intermedia* . In contrast, pMMR tumors exhibit a lower overall bacterial abundance, although they still show enrichment of *F. nucleatum* and *Selenomonas sputigena* [166, 167]. Further analysis of the relationship between tumor microbiota and clinical molecular markers or prognostic subtypes identified microbial community subtypes enriched with *F. nucleatum* , *Leptotrichia hofstadii* , and *Centipeda periodontii* as being associated with the majority of dMMR CRC cases [168]. The presence of microorganisms within tumors underscores the significance of the microbiota, suggesting that this characteristic may be leveraged in the future to directly target tumors through microbiome‐based approaches or to deliver antitumor drugs aimed at modulating the TME [169].

Currently, most research in related fields primarily focuses on exploring the impact of the gut microbiome on immunotherapy treatment responses across various cancer types from a macro perspective or on summarizing the mechanisms of microbiota in CRC and investigating how gut microbiota modulate the host's antitumor immune response. However, this often overlooks the integration of differences in immunotherapy efficacy among different types of CRC, such as pMMR and dMMR, and their relationship with gut microbiota. Therefore, this review aims to conduct a systematic study that spans different types of CRC, the TME, and the differences in gut microbiota and their interactions. It is expected to provide a significant theoretical basis for enhancing the efficacy of CRC immunotherapy or optimizing combination therapy strategies. Nevertheless, substantial efforts are still needed to deepen our understanding of the mechanistic connections and to translate these findings into clinical practice.

## Author Contributions

**Fayuan Wang:** writing – original draft, conceptualization. **Weidong Chen:** writing – original draft, methodology. **Yingtian Jia:** writing – original draft. **Tao He:** writing – original draft. **Siyi Wu:** writing – original draft. **Jiahui Xiang:** visualization. **Rui Chen:** visualization. **Qingfeng Jiang:** visualization. **Tengjiang Yu:** writing – original draft, software. **Yong Lan:** software, data curation. **Wusheng Li:** project administration, data curation. **Liang Ma:** writing – review and editing. **Ping He:** writing – review and editing. **Shichao Li:** writing – review and editing.

## Disclosure

All claims expressed in this article are solely those of the authors and do not necessarily represent those of their affiliated organizations, or those of the publisher, the editors, and the reviewers. Any product that may be evaluated in this article, or claim that may be made by its manufacturer, is not guaranteed or endorsed by the publisher.

## Ethics Statement

The authors have nothing to report.

## Consent

The authors have nothing to report.

## Conflicts of Interest

The authors declare no conflicts of interest.

## Data Availability

Data sharing not applicable to this article as no datasets were generated or analysed during the current study.
